# Heterosis-associated genes confer high yield in super hybrid rice

**DOI:** 10.1007/s00122-020-03669-y

**Published:** 2020-08-27

**Authors:** Tianzi Lin, Cong Zhou, Gaoming Chen, Jun Yu, Wei Wu, Yuwei Ge, Xiaolan Liu, Jin Li, Xingzhou Jiang, Weijie Tang, Yunlu Tian, Zhigang Zhao, Chengsong Zhu, Chunming Wang, Jianmin Wan

**Affiliations:** 1 State Key Laboratory of Crop Genetics and Germplasm Enhancement, Nanjing Agricultural University, Nanjing, 210095 China; 2 National Key Facility for Crop Gene Resources and Genetic Improvement, Institute of Crop Science, Chinese Academy of Agricultural Sciences, Beijing, 100081 China; 3 Zhenjiang Institute of Agricultural Sciences in Hilly Region of Jiangsu Province, Jurong, 212400 China; 4 Department of Immunology, The University of Texas Southwestern Medical Center, Dallas, TX 75390 USA; 5 Key Laboratory of Biology, Genetics and Breeding of Japonica Rice in the Mid-lower Yangtze River, Ministry of Agriculture, Nanjing, 210095 China; 6 Jiangsu Plant Gene Engineering Research Center, Nanjing, 210095 China; 7 Jiangsu Collaborative Innovation Center for Modern Crop Production, Nanjing, 210095 China

## Abstract

**Key message:**

Heterosis QTLs, including *qSS7* and *qHD8,* with dominance effects were identified through GBS and large-scale phenotyping of CSSLs and hybrid F_1_ populations in a paddy field.

**Abstract:**

Heterosis has contributed immensely to agricultural production, but its genetic basis is unclear. We evaluated dominance effects by creating two hybrid populations: a B-homo set with a homozygous background and heterozygous chromosomal segments and a B-heter set with a heterozygous background and homozygous segments. This was achieved by crossing a set of 156 backcrossed-derived chromosome segment substitution lines (CSSLs) with their recurrent parent (9311), the male parent of the first super-high-yield hybrid Liangyoupei9 (LYP9), and with the female parent (PA64s) of the hybrid. The CSSLs were subjected to a genotyping-by-sequencing analysis to develop a genetic map of segments introduced from the PA64s. We evaluated the heterotic effects on eight yield-related traits in the hybrid variety and F_1_ populations in large-scale field experiments over 2 years. Using a linkage map consisting of high-density SNPs, we identified heterosis-associated genes in LYP9. Five candidate genes contributed to the high yield of LYP9, with *qSS7* and *qHD8* repeatedly detected in both B-hybrid populations. The heterozygous segments harboring *qSS7* and *qHD8* showed dominance effects that contributed to the heterosis of yield components in the hybrid rice variety Liangyoupei9.

**Electronic supplementary material:**

The online version of this article (10.1007/s00122-020-03669-y) contains supplementary material, which is available to authorized users.

## Introduction

Rice is a major cereal crop, and hybrid rice has significantly contributed to yield improvement in China. Genes responsible for heterosis of yield have been identified in the widely grown, two-line hybrid variety Liangyoupei9 (LYP9, cross PA64s/9311) (Li et al. 2016).

A group of heterosis-related genes was identified by correlating the concurrence of differentially expressed genes and yield-related QTL (quantitative trait loci) (Wei et al. 2009). The yield-related QTL *qSN8* was confirmed as *DTH8* (*days to heading 8*) by a complementation test (Gao et al. 2013). This major QTL for yield heterosis was also found to have pleiomorphic effects and was designated as *DTH8*/*Ghd8*/*LHD1* as a result of integrating genetics and omics analyses (Li et al. 2016). By positional cloning, *pms1* (photoperiod-sensitive genic male sterility) was isolated, and it was found to encode phasiRNAs that are involved in rice development (Fan et al. 2016). However, it is unclear if the heterozygous alleles of these genes affect heterosis with dominance effects.

A total of 66 chromosome segment substitution lines (CSSLs) and their corresponding F_1_ hybrids to the recurrent parent were genotyped by 137 SSR markers (Wang et al. 2012) to identify three major stable QTLs for heterosis in six environments. The QTL *qFCC7*_*L*_ controlling the chlorophyll and photosynthetic rate was found to underlie low nitrogen stress tolerance in rice. This was based on 132 recombinant inbred lines (RILs) and fine mapping of the BC_4_F_2_ population derived from a cross between PA64s and 9311 (Ye et al. 2017). Forty-six QTLs associated with yield were detected using a set of 156 chromosome segment substitution lines (CSSLs) with overlapping segments from PA64s in a genetic background of 9311 (Liu et al. 2016). Neither population size nor the number of molecular markers in that study was sufficient for gene isolation and the analysis of gene effects.

To understand the mechanisms underlying heterosis in rice, we used 156 CSSLs previously developed by Liu et al. (2016). The CSSLs represented a population of backcross-derived lines of high-yielding variety 9311 with the elite variety Pei’ai64s (PA64s) as the donor. These varieties were also the parents of hybrid variety Liangyoupei9 (LYP9, cross PA64s/9311). We identified 10,768 SNPs in the CSSL population through genotyping-by-sequencing (GBS), and we developed two sets of hybrid populations to study heterotic effects. The B-homo hybrid set involved crosses of the CSSLs to the recurrent parent and had predominantly heterozygous substituted segments and a homozygous 9311 background. The B-heter hybrid set, produced using crosses to the donor parent, was homozygous for the substituted fragments and heterozygous for the genetic background. Heterosis of eight yield-related traits was evaluated over 2 years by comparing F_1_ sets with recurrent parent or Liangyoupei9 (LYP9).

We report here that *qSS7* and *qHD8* play key roles in the rate of seed setting and the heading date in a heterozygous background, respectively. Distinguished from other heterosis QTLs, *qSS7* and *qHD8* have pleiotropic effects that confer stable and strong heterosis in hybrid rice.

## Materials and methods

### CSSL and derived F_1_ populations

We developed 156 chromosome segment substitution lines (CSSLs) from a cross between high-yield cultivar 9311 as the recurrent parent and an elite cultivar Pei’ai64s (PA64s) as the donor. To understand the genetic basis of yield-related traits of LYP9, we developed two F_1_ populations: a B-homo F_1_ set derived from CSSLs/9311 with a heterozygous segment in a homozygous background and a B-heter F_1_ set derived from PA64s/CSSLs with a homozygous segment in a heterozygous background, respectively. Two F_1_ sets corresponding to CSSLs, background homozygous with segment heterozygous, abbreviated as B-homo F_1_, were produced from CSSLs/9311, and background heterozygous with segment homozygous, abbreviated as B-heter F_1_, were produced from PA64s/CSSLs.

The two parents, CSSLs, B-homo F_1_ (homozygous background with heterozygous segment) and B-heter F_1_ (heterozygous background with homozygous segment), were grown at Nanjing in 2016 and 2017, and the trials were designated as Nanjing environments E1 and E2, respectively. Each entry was a plot of two rows, containing 10 individual plants in a randomized block design with two replications, but with each parental CSSL and its corresponding F_1_ hybrid being planted side by side. Individual plant and row spacing within plots was 16.5 cm, and plot spacing was 23.5 cm. Fertilizer levels and disease control followed local recommendations.

### Plant phenotyping

Heading date (HD) were recorded, and six plants per line were harvested from each plot at maturity. Plant height (PH), effective panicle number (EPN), grain number per panicle (GN), rate of seed setting (SS), 1000-grain weight (TGW), and panicle length (PL) were determined on three plants from each plot. Yield per plant (YPP) was measured and calculated as the average weight per plant of bulked grain obtained from the six plants. The measuring procedures were described in Xiao et al. (1998).

### SNP genotyping-by-sequencing

Genotyping-by-sequencing (GBS) (Poland et al. 2012) of each entry was used for SNP calling. DNA for the GBS library was extracted from 14-d-old seedlings. *Pst*I and *Msp*I (NEB) were used for digestion and T4 ligase (NEB) for ligation. All of the samples were pooled for purification and PCR-amplification. The library was sequenced using the Illumina Platform, and the data were analyzed by Tassel software (Glaubitz et al. 2014).

### Data analysis and QTL mapping

Estimates of marker distances, chromosome lengths, substituted segments, and overall genome size were based on the linkage map (Moncada et al. 2001). Construction of graphical genotypes and calculation of percentage of donor genome in each CSSL were performed using Tassel software. If two neighboring loci had alleles from the donor parent, then the interval between them was considered to be the length of the segment. If one locus had an allele from the recurrent parent and the other locus had the allele from the donor parent, then half of the interval between them was considered to represent the length of the substitution.

**Fig. 1:**
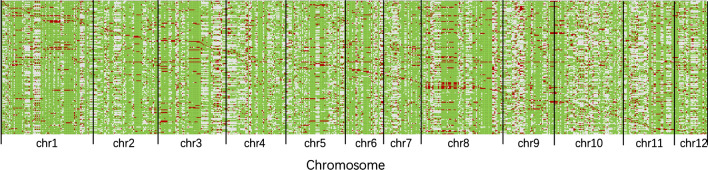
SNP map of the CSSL populations. SNPs were detected by genotyping-by-sequencing and analyzed using Tassel software. The green background of the SNP map is from 9311, the red substitution segments are from PA64s, and the gray segments are heterozygous or undetected

QTL in CSSLs and their F_1_ hybrid sets were detected by QTL IciMapping software (Li et al. 2007; Wang et al. 2007) based on the combined analysis of their marker genotypic and phenotypic data. Based on the map (Fig. 1) and three sets of phenotypic data (a set of CSS lines and corresponding hybrid sets in two environments), the effects of chromosome segments from the donor parent in CSSL and heterozygous genotypes in F_1_ sets were analyzed. The LOD threshold, a measure of significance, was set at 5.0 (Wang et al. 2006). QTL nomenclature followed McCouch et al. (1997). The estimated additive and dominance effects were used to calculate |d/a| and thereby classify the QTL as additive (A) (|*d*/*a*| < 0.2), partially dominant (PD) (0.2 ≤ |*d*/*a*| < 0.8), completely dominant (CD) (0.8 ≤ |*d*/*a*| < 1.2), or overdominant (OD) (|*d*/*a*| ≥ 1.2) (Stuber et al. 1992). Heterosis in the B-homo set was expressed as the mid-parent heterosis value: B-homo F_1_ - (CSSL mean + 9311)/2.

## Results

### Yield-related traits in CSSLs and F_1_ hybrids

We constructed two F_1_ sets, B-homo F_1_ and B-heter F_1_, based on a set of 156 CSSLs (Fig. 1). Heterosis of the eight yield-related traits was evaluated by comparing the F_1_ lines with two parents or Liangyoupei9 (LYP9) in two environments (Fig. S1).

The data for parental cultivar 9311, CSSLs, and corresponding F_1_ sets are given in Table S1. PL, PH, EPN, SS, TGW, and YPP showed positive heterosis (measured as MPH, mid-parent heterosis) in the B-homo F_1_ set across both environments, but only EPN showed positive heterosis in the B-heter F_1_ set. HD, PL, SS, and YPP showed negative heterosis (measured as ES, effects of substituted segment) in the B-heter F_1_ set in both environments. Correlation analysis of two sets of F_1_ traits and CSSL traits, based on the mean values of eight yield-related traits of F_1_ hybrid sets across environments, showed that yield per plant (YPP) of the B-homo set was not significantly related to the eight traits of CSSLs. This indicated that the mid-parent heterosis of yield per plant (YPP) mainly came from the heterozygous effect of QTL for yield traits in a homozygous background. However, YPP in the B-heter set was positively related to the HD and YPP of the CSSLs and significantly related to PH, SS, and TGW of CSSLs. This indicated that the yield level of the hybrid was due to higher YPP, PH, SS, and TGW and the prolonged growth period relative to the parental lines (Table S2). Combined ANOVA of yield across environments showed that the yield component traits were influenced by significant genotypic and environmental effects, as well as genotype–environment interactions (Table S3).

### SNP genotyping-by-sequencing and QTL mapping

We identified 10,768 SNPs in CSSLs subjected to the GBS, which we also applied in our previous studies with minor modifications (Tang et al. 2016, 2019; Yu et al. 2020). The SNPs were evenly distributed across the 12 chromosomes (Fig. S2). We detected QTLs underlying the yield of hybrids by association of SNPs with trait phenotypes in QTL analyses. QTLs were detected using QTL IciMapping software (Meng et al. 2015), which is available on (http://www.isbreeding.net/software/). We used the ICIM (Inclusive Composite Interval Mapping) method for QTL identification (Wang 2009). We chose the LOD 5.0 as the threshold for QTL analysis. The QTLs were listed when they were detected in both environments or two F_1_ sets (Table 1). The identified QTLs were named according to McCouch et al. (1997).

**Table 1:** Five QTLs were identified in the B-homo and the B-heter F_1_ sets

QTL	Trait name	Chr	Left marker	Right marker	LOD	PVE (%)	Add	Dom	$\left| {\frac{d}{a}} \right|$
*qSS4*	SS-He-1	4	17,868,628	18,154,552	11.38	5.977	− 8.551	7.678	0.898
	SS-He-2	4	17,868,628	18,154,552	9.62	6.546	− 8.795	7.430	0.845
*qPH6*	PH-He-1	6	26,412,872	26,593,549	10.20	14.098	− 4.424	5.149	1.164
	PH-He-2	6	26,412,872	26,593,549	16.46	18.999	− 5.509	7.790	1.414
*qSS9*	SS-He-1	9	21,383,583	21,652,040	15.25	8.731	− 12.741	− 1.542	0.121
	SS-He-2	9	21,383,583	21,652,040	10.40	7.417	− 9.078	1.352	0.149
*qSS7.1*	SS-Ho-1	7	4,272,700	4,499,836	4.36	9.043	6.678	− 10.755	1.611
	PH-He-2	7	4,272,700	4,499,836	7.83	7.900	− 4.544	− 5.078	1.118
	SS-He-2	7	4,272,700	4,499,836	42.51	49.647	− 19.877	19.032	0.957
	YPP-He-1	7	4,272,700	4,499,836	16.71	33.341	− 9.788	9.402	0.961
	YPP-He-2	7	4,272,700	4,499,836	17.02	26.124	− 8.987	8.635	0.961
*qHD8*	HD-Ho-1	8	4,094,492	4,308,948	64.59	76.165	6.096	− 5.893	0.967
	HD-Ho-2	8	4,094,492	4,308,948	59.16	75.513	− 8.075	7.525	0.932
	PH-Ho-2	8	4,094,492	4,308,948	23.21	31.673	7.724	− 9.389	1.216
	PH-Ho-1	8	4,094,492	4,308,948	14.65	32.693	7.215	− 10.430	1.446
	YPP-Ho-1	8	4,094,492	4,308,948	4.63	11.491	4.044	− 5.258	1.300
	GN-Ho-1	8	4,094,492	4,308,948	4.43	12.181	19.276	− 21.863	1.134
	HD-He-1	8	4,094,492	4,308,948	59.16	76.477	− 8.075	7.525	0.932
	HD-He-2	8	4,094,492	4,308,948	43.75	43.303	− 8.367	8.258	0.987
	PH-He-1	8	4,094,492	4,308,948	15.25	22.828	− 6.261	1.453	0.232
	GN-He-1	8	4,094,492	4,308,948	9.91	16.630	− 27.897	34.233	1.227
	TGW-He-2	8	4,094,492	4,308,948	7.10	13.084	− 1.049	2.203	2.101

*qSS7.1* and *qHD8* were identified repeatedly in both homozygous and heterozygous backgrounds

*SS* rate of seed setting, *PH* plant height, *HD* heading date, *YPP* yield per plant, *GN* grain number per panicle, *TGW* 1000-grain weight, *LOD* log of odds, *PVE* phenotypic variation explained, *Add* additive effect, *Dom* dominance effect, $\left| {\frac{d}{a}} \right|$, dominance ratio, the absolute value of dominance effect to additive effect

### QTL analysis for yield-related traits in *LYP9*

In a homozygous background, 14 QTLs with dominance effects were identified at the threshold of LOD 5.0 (Table S4). Five showed overdominance, six were completely dominant, and three were partially dominant. No QTL had an additive effect in a predominantly homozygous background, indicating that dominance effects play an important role in heterosis.

A total of 28 QTLs were detected in a heterozygous background, indicating that dominance affects contributed to heterosis (Table S5). QTLs *qHD8* and *qSS7* were identified as pleiotropic traits in both F_1_ sets (Table 1). The *qHD8* overlapped the position of gene *DTH8* on chromosome 8, whereas the *qSS7* association with SS, PH, and YPP was novel.

Heading date (HD) is an important agronomic trait in hybrid rice, and it affects the desired yield level in rice breeding. QTL *qHD8,* controlling heading date, was a highly significant locus explaining about 60% of the phenotypic variation. PH is a target trait for hybrid rice improvement. In this study, *qPH5*, *qPH6*, *qPH7*, and *qPH9* and *qPH6* explained 14.10 and 19.00% of the phenotypic variation in each environment. SS is also an important trait in heterosis, and *qSS3.1*, *qSS3.2*, *qSS4*, *qSS6*, *qSS7.1*, *qSS7.2*, *qSS9,* and *qSS10* were detected in both environments, explaining 5.98–6.55%, and 7.41–8.73% of the phenotypic variation. For GN, YPP, EPN, and TGW, we detected *qGN8*, *qYPP3*, *qYPP7* (*qSS7*), *qYPP9*, *qEPN4*, and *qTGW8* (*qHD8*). Most of the QTLs controlled a single trait, but *qSS7.1* and *qHD8* were exceptions in having pleiotropic effects on PH, SS, and YPP, and HD, PH, GN, and TGW, respectively.

### Heterosis-related loci and candidate genes

**Fig. 2:**
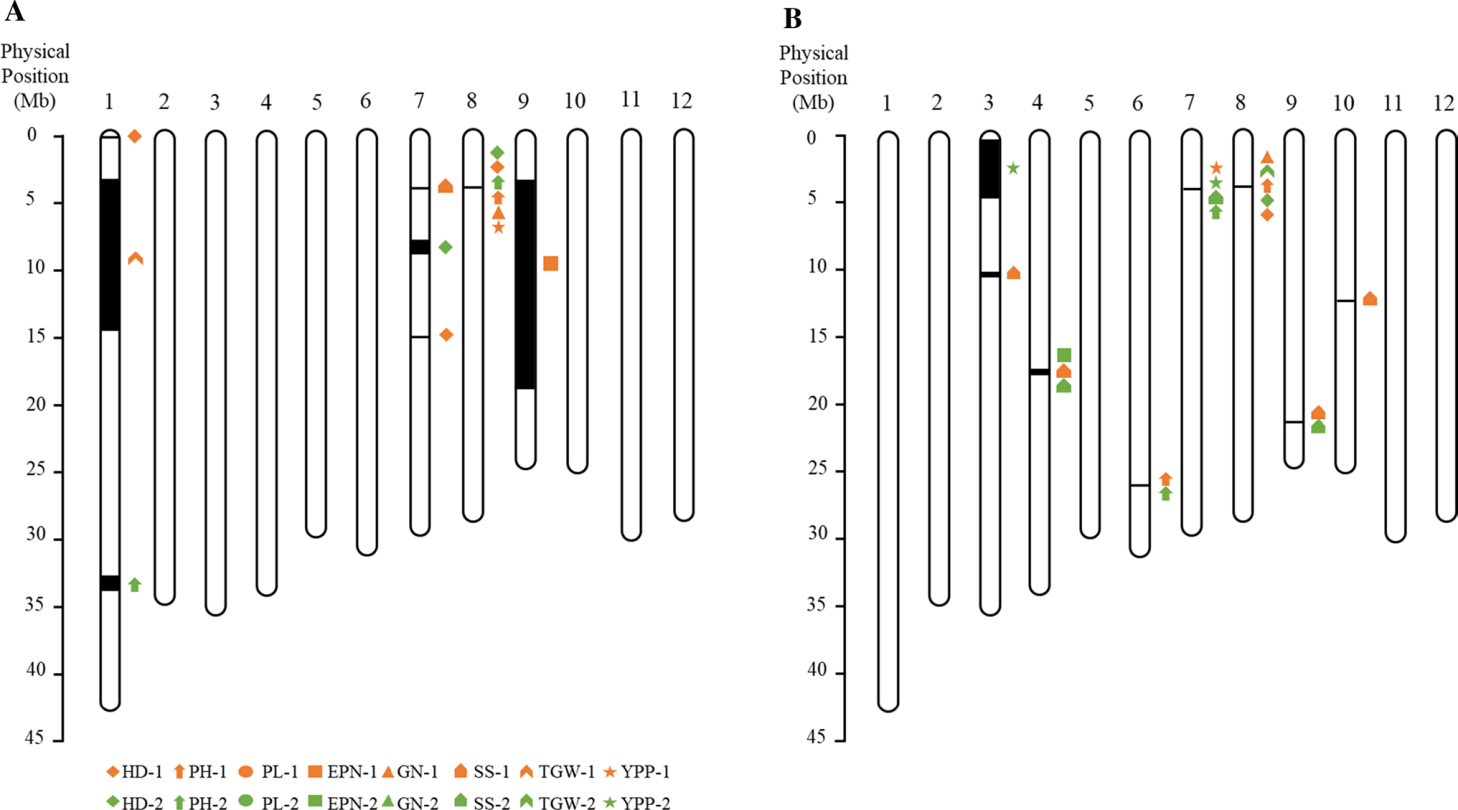
QTLs detected on 12 chromosomes. A scale at the left side represents physical position (Mb). QTLs detected in the environment E1 were marked in orange, and QTLs in E2 were marked in green. Among these QTLs, *qSS7* and *qHD8* were identified repeatedly in the two hybrid sets in a homozygous background (**a**) and heterozygous background (**b**), respectively

We focused on a detailed study of QTLs *qSS7* and *qHD8* (Fig. 2, Fig. S3). *qHD8* was previously reported to affect heterosis based on SN (spikelet number per panicle) (Gao et al. 2013; Li et al. 2016), heading date, and plant height (Li et al. 2016).

**Fig. 3:**
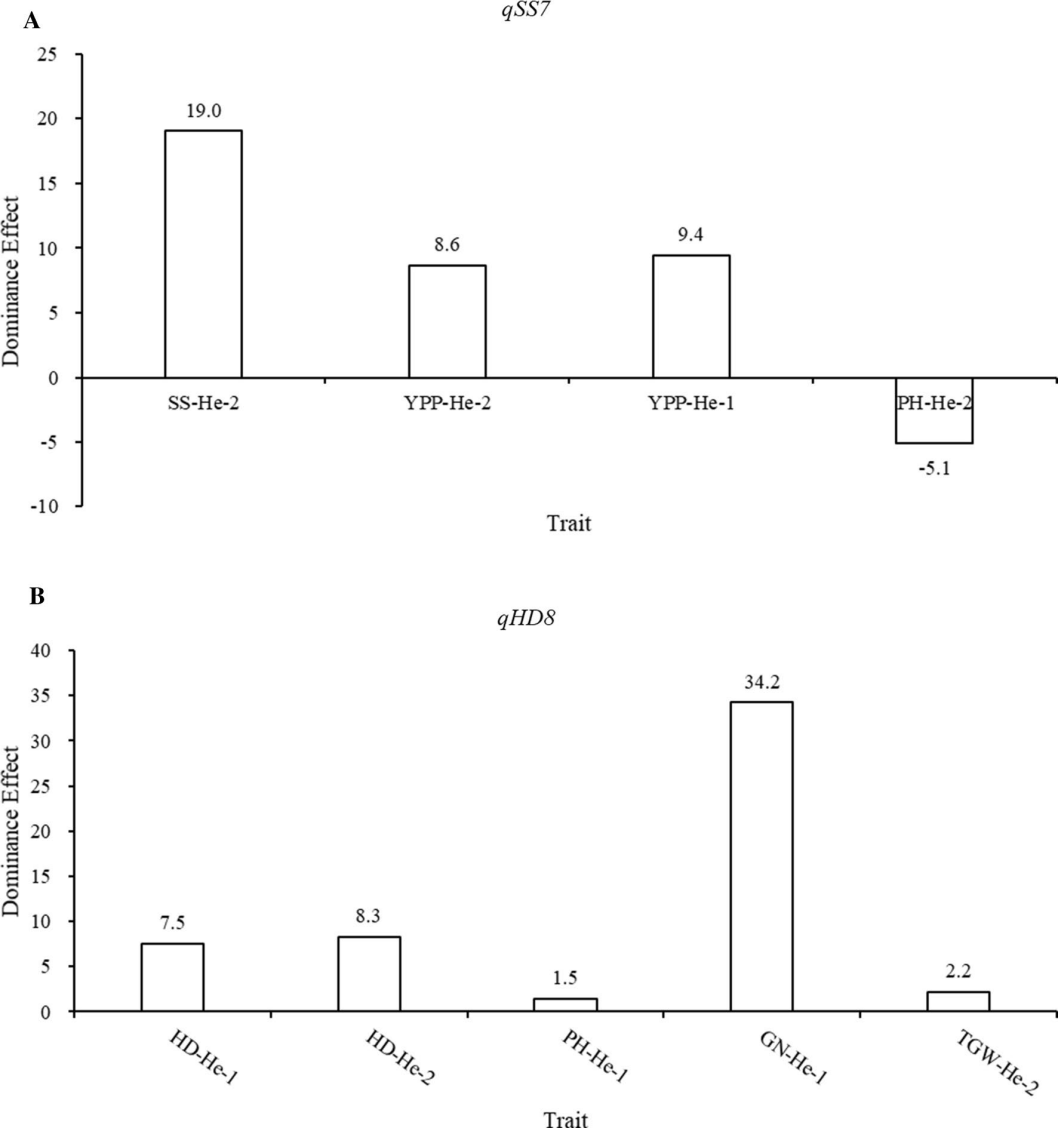
Dominance effects of the two QTLs *qSS7* and *qHD8* in a heterozygous background. **a** QTL *qSS7* shows positive values in terms of SS-2, YPP-1 and YPP-2 traits, and negative values in PH-2. **b** QTL *qHD8* shows positive values in terms of HD-1, HD-2, PH-1, GN-1 and TGW-2 traits

We analyzed the dominance effects of the two QTLs in heterozygous backgrounds, and both showed positive heterosis potential of heterozygous segments (Fig. 3). The dominance effects of *qSS7* on SS, YPP, and PH were 19.0, 9.4, and − 5.1, respectively. Therefore, heterozygosity of *qSS7* conferred heterosis by prolonging the growth period and reducing plant height (Fig. 3a). Similarly, the dominance effects of *qHD8* on HD, PH, GN, and TGW were 8.3, 1.5, 34.2, and 2.2, respectively (Fig. 3b).

### Heterosis utilization potentials

**Fig. 4:**
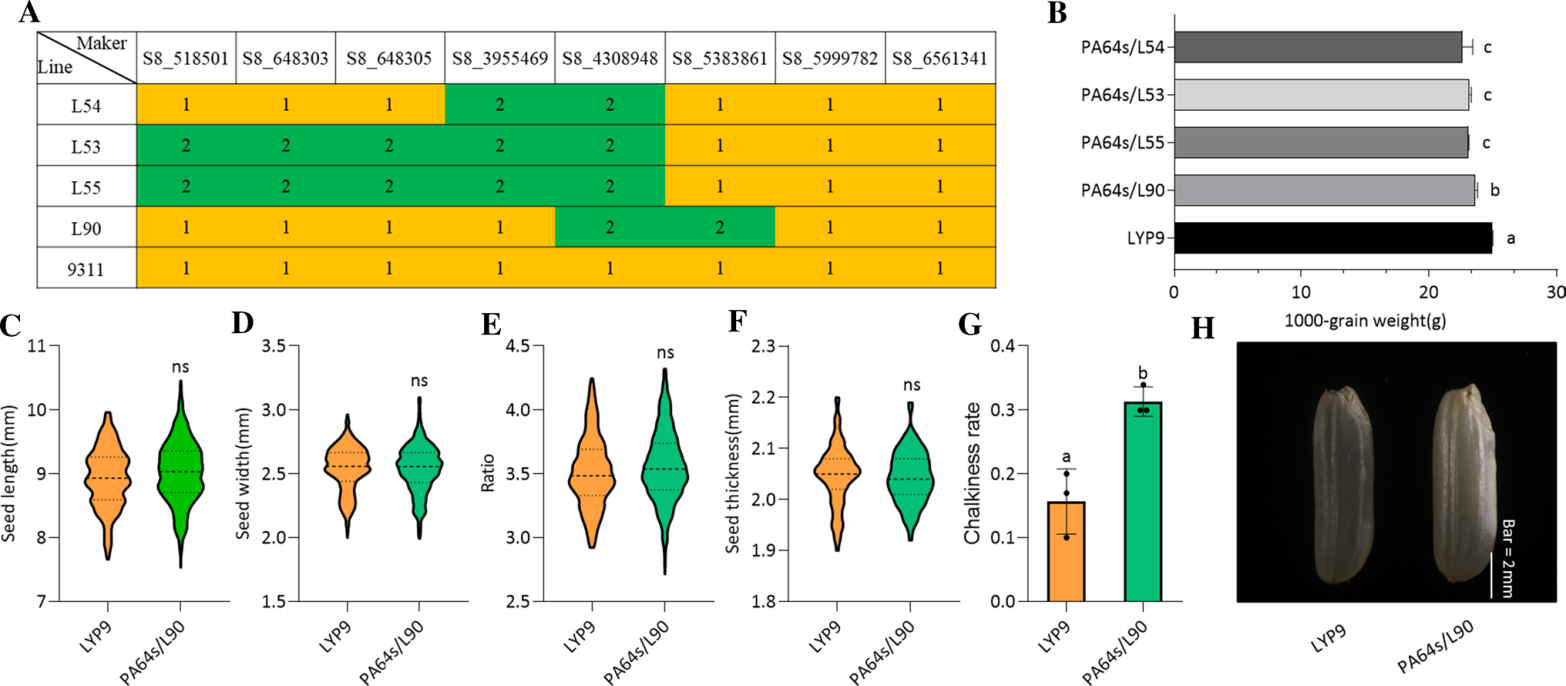
Comparisons of F_1_ lines derived from PA64s/L54, PA64s/L53, PA64s/L55, PA64s/L90, and LYP9 lines. L54, L53, L55, and L90 were four CSSLs. **a** The physical map of the 9311 variety and the CSSLs harboring *qHD8*^PA64s^. **b** Comparison of 1000-grain weight of the four F_1_ lines and LYP9. **c**–**g** The seed length, width, length, width ratio, and level of chalkiness in LYP9 and PA64s/L90. **h** Image of the seeds of LYP9 and PA64s/L90

We found that *qHD8*^9311^ increased TGW (Fig. 4b). The homozygous fragment of *qHD8*^PA64s^ in a heterozygous background conferred lower TGW as shown in lines L54, L53, L55, and L90 (Fig. 4a, b). To determine why these lines had decreased TGW, we measured the seed length, width, length width ratio, and thickness, and we found that PA64s/L90 (homozygous *qHD8*^PA64s^) was not significantly different from LYP9 (Fig. 4c–f). However, a higher chalkiness rate was found in PA64s/L90 compared to LYP9, and it probably decreased TGW (Fig. 4g, h).

**Fig. 5:**
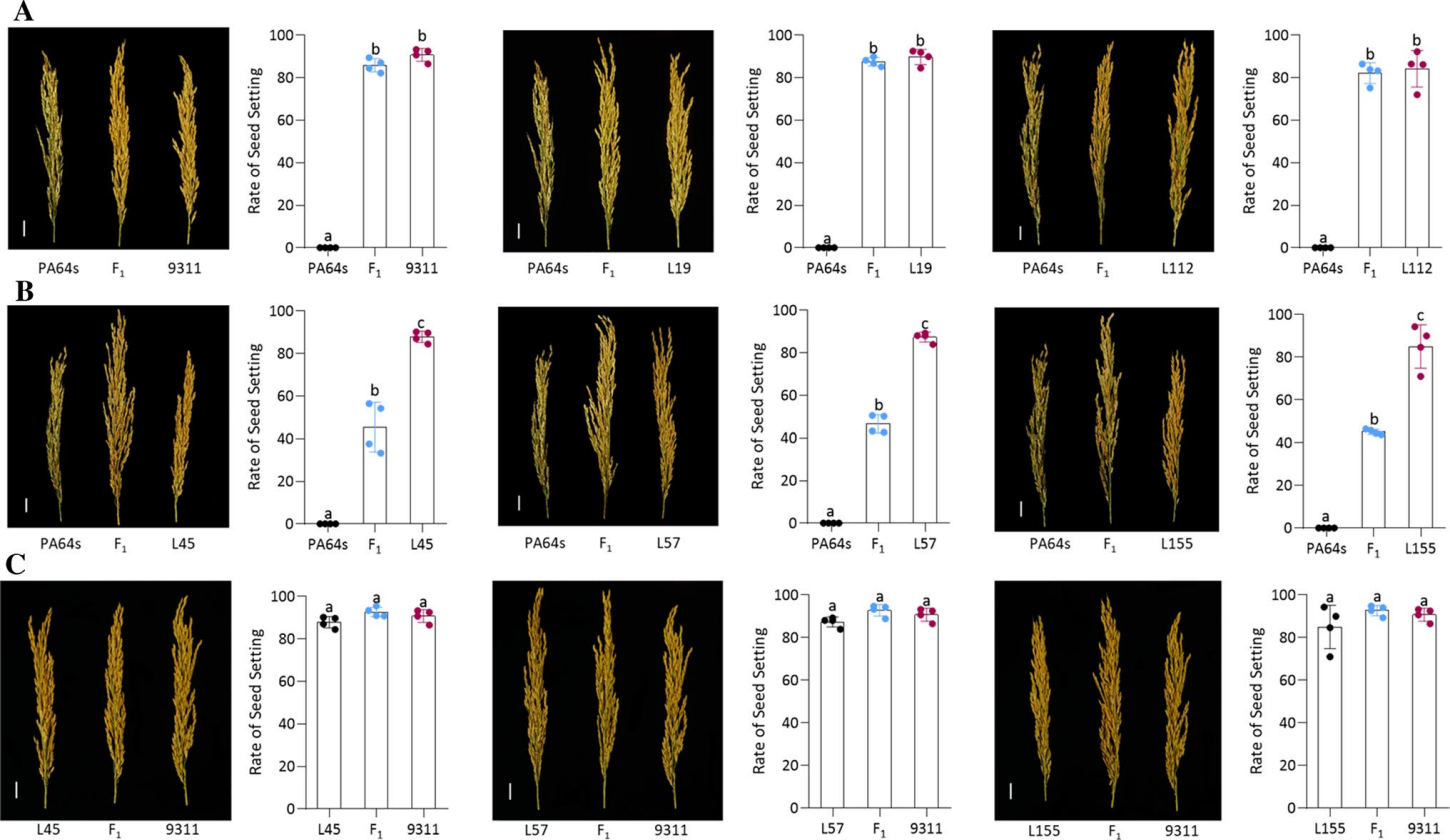
Rates of seed setting of the lines with *qSS7*^PA64s/9311^ and *qSS7*^PA64s/PA64s^ in different backgrounds. L19, L112, L45, L57, and L155 were the five CSSLs from the QTL mapping population. *qSS7*^PA64s/9311^ represents the heterozygous segment of the *qSS7*, and *qSS7*^PA64s/PA64s^ represents the homozygous segment of the *qSS7*. **a** Rates of seed setting of F_1_ harboring *qSS7*^PA64s/9311^ in a heterozygous background and parents. Left: comparison of PA64s, PA64s/9311 and 9311; middle: comparison of PA64s, PA64s/L19 and L19; right: comparison of PA64s, PA64s/L112 and L112. **b** Rates of seed setting of F_1_ harboring *qSS7*^PA64s/PA64s^ in a heterozygous background and parents. Left: comparison of PA64s, PA64s/L45 and L45; middle: comparison of PA64s, PA64s/L57 and L57; right: comparison of PA64s, PA64s/L155 and L155. **c** Rates of seed setting of F_1_ harboring *qSS7*^PA64s/9311^ in a homozygous background and parents. Left: comparison of L45, L45/9311 and 9311; middle: comparison of L57, L57/9311 and 9311; right: comparison of L155, L155/9311 and 9311

The QTL *qSS7* had effects on PH, SS, and YPP with LOD values ranging from 7.83 to 42.51 and PVE from 7.90 to 49.65%. A phasiRNA was reported to control the rate of seed setting under long-day conditions (Fan et al. 2016). The SS of F_1_ with *qSS7*^PA64s/9311^ in a heterozygous background was not significantly different from 9311 or the male parent (Fig. 5a). However, the SS of F_1_ with *qSS7*^PA64s/PA64s^ in a heterozygous background was significantly lower than the male parent (Fig. 5b), whereas the SS of F_1_ with *qSS7*^PA64s/PA64s^ in homozygous background was not significantly different from the parents (Fig. 5c). Interestingly, the QTL was also detected to underlie PH in the B-heter F_1_ set, although phasiRNAs have not been previously reported to control PH.

### Heterozygous segments lead to superior performance in B-heter set

**Fig. 6:**
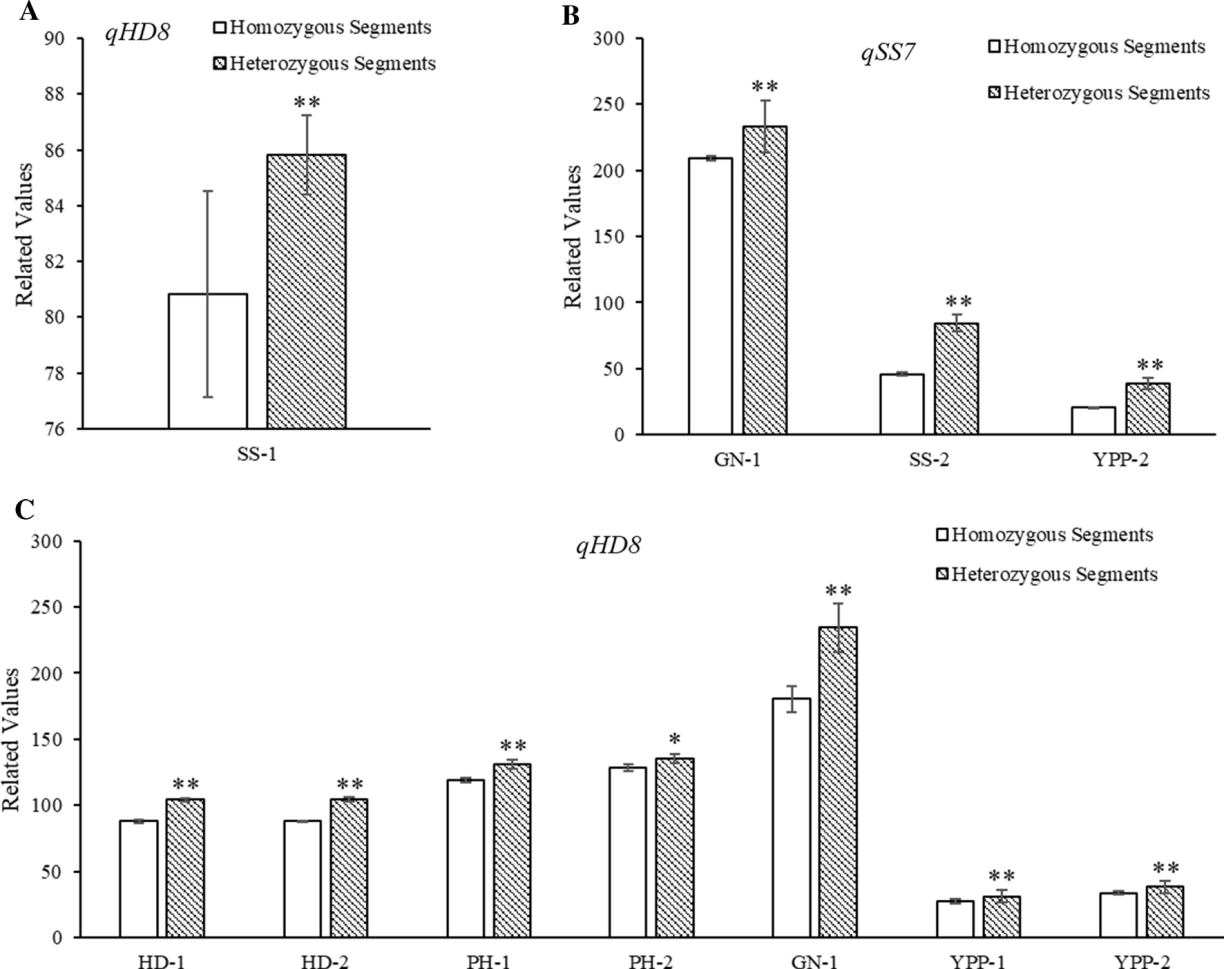
Comparisons of phenotypic variances for the two QTLs in the F_1_ sets. **a** Comparison of the SS-1 trait of the B-homo F_1_ sets with the homozygous and heterozygous *qHD8* segments, respectively. **b** Comparisons of the GN-1, SS-2 and YPP-2 traits of the B-heter F_1_ sets with the homozygous and heterozygous *qSS7* segments, respectively. **c** Comparisons of the HD-1, HD-2, PH-1, PH-2, GN-1, YPP-1 and YPP-2 traits of the B-heter F_1_ sets with the homozygous and heterozygous *qHD8* segments, respectively. No significant differences were observed when comparing traits of the B-homo F_1_ sets with the homozygous and heterozygous *qSS7* segments. *PH* plant height, *SS* rate of seed setting, *GN* grain number per panicle, *YPP* yield per plant, *HD* heading date. *, **P < 0.05 and 0.01, respectively

The GBS analyzed SNPs that identified *qSS7* and *qHD8* in the CSSLs. An A/G SNP is located near *qSS7*, whereas adjacent SNPs CC/TA were close to *qHD8*. Using these SNPs as markers, we classified the CSSLs into four groups, *qSS7*^9311^/*qHD8*^9311^ (A/CC), *qSS7*^9311^/*qHD8*^PA64s^ (A/TA), *qSS7*^PA64s^/*qHD8*^9311^ (G/CC), and *qSS7*^PA64s^/*qHD8*^PA64s^ (G/TA), and we analyzed the phenotypic data of two loci in both F_1_ sets. The heterozygous *qHD8* segment in a homozygous background significantly increased SS, but the homozygous *qHD8* did not (Fig. 6a). Heterozygous *qSS7* in a heterozygous background (equal to LYP9) significantly increased GN-1, SS-2, and YPP-2, but the homozygous *qSS7* did not (Fig. 6b). Similarly, the heterozygous *qHD8* in a heterozygous background increased HD-1, HD-2, PH-1, PH-2, GN-1, YPP-1, and YPP-2 of hybrid set, but the homozygous *qHD8* did not (Fig. 6c). These results indicated that heterozygous segments of the two QTLs conferred superior performances, especially in B-heter set.

### Interaction between *qSS7* and *qHD8*

**Fig. 7:**
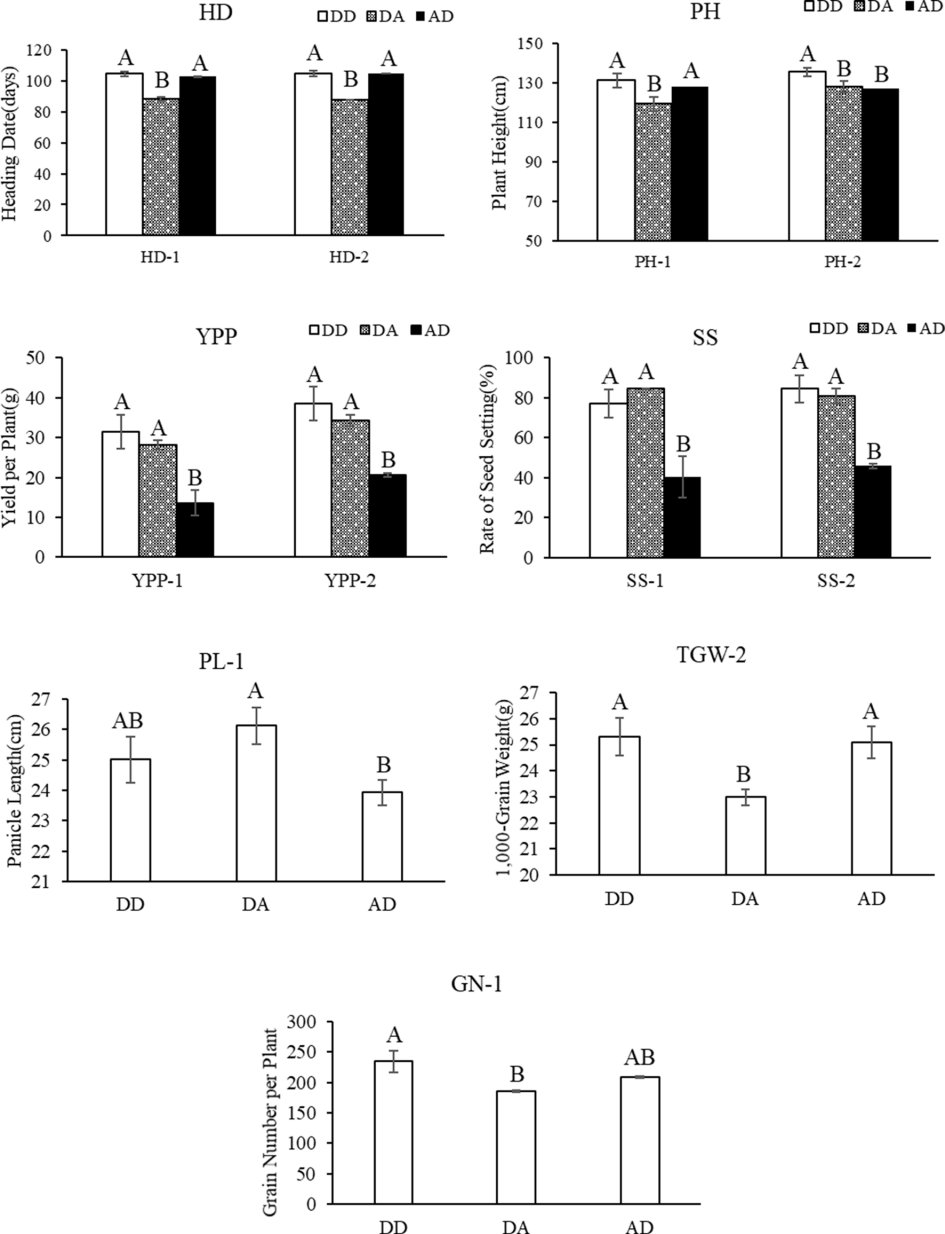
Multiple comparisons of interactions between the two QTLs *qSS7* and *qHD8* in the B-heter F_1_ sets. DD represents dominance–dominance interaction between *qSS7*^PA64s/9311^/*qHD8*^PA64s/9311^; DA represents dominance–additive interaction between *qSS7*^PA64s/9311^/*qHD8*^PA64s/PA64s^; AD represents additive–dominance interaction between *qSS7*^PA64s/PA64s^/*qHD8*^PA64s/9311^. *HD* heading date, *PH* plant height, *PL* panicle length, *GN* grain number per panicle, *SS* rate of seed setting, *TGW* 1000-grain weight, *YPP* yield per plant. Different uppercase letters indicate significance at *P* < 0.01

Analysis of variance (Table S6) showed significant interactions between dominance effects of the two QTLs. There were significant differences among all of the combinations of the two QTLs showing that dominance–dominance interactions contributed to the superior performance of *qSS7*^PA64s/9311^/*qHD8*^PA64s/9311^ heterozygotes in the B-heter F_1_ set that were labelled as DD with the highest performances in the eleven traits (Fig. 7).

## Discussion

Heterosis is defined as the superior performance of a hybrid over the parental mean. Understanding the genetics of heterosis should allow production of hybrid varieties by molecular design breeding. To analyze the mechanism of heterosis at the genetic level, four conditions are required: (1) genetic materials with strong heterosis as demonstrated by field trials; (2) specific agronomic traits defined; (3) a suitable experimental population to analyze the genetic components of heterosis, including dominance, overdominance, and unbiased estimation of epistasis; and (4) a high-density genetic linkage map for QTL analysis. In this study, we identified heterosis-associated loci using 156 CSSLs derived from the parents of a super hybrid rice variety crossed to their corresponding parents. This enabled the study of specific introgressions, in homozygous and heterozygous conditions, against the genetic backgrounds that were, in turn, heterozygous or homozygous. Phenotypic variation from a field trial repeated in 2 years was then regressed on a genetic map consisting of 10,768 SNP markers. Two major QTLs were chosen for detailed study. These were *qSS7*, conferring a high rate of seed setting and low plant height, and *qHD8*, with pleiotropic effects on HD, PH, GN, and TGW.

Heterosis includes additive effects, dominance effects, and epistatic effects from the interaction effects of non-alleles between loci (Hua et al. 2003). The study of epistatic effects is challenging for the following reasons. To detect the potential interaction effects of *n* loci, there are *n(n-1)/2* combinations of loci of which there are four types of interaction modes. These include additive–additive interaction, additive–dominance interaction, dominance–additive interaction, and dominance–dominance interaction. Interaction effects are also susceptible to genetic background interference and environmental effects. Therefore, the study of epistatic effects requires specific genetic cross designs and an appropriate genetic background of test materials. Although the hybrid F_1_ of the CSSL-based NCII design combined with BCF_1_ or TCF_1_ populations can eliminate genetic background interference and facilitate statistical analysis, the workload is substantial. The materials of B-heter F_1_ set generated in this study enabled the evaluation of four types of interaction modes, i.e., AA (additive–additive interaction), AD (additive–dominance interaction), DA (dominance–additive interaction) and DD (dominance–dominance interaction). We focused on analyzing the effects of substituted segments, deletion of the dominance effects of the fragment, and the dominance–dominance interaction between the fragments. To achieve the genetic response for heterosis utilization and make full use of advantageous allelic mutations directly, the targeted fragments with disadvantageous heterotic loci were replaced.

We collected the phenotypic data of a set of CSSLs and corresponding F_1_ hybrid sets in two environments. Based on a SNP map and phenotypic data of eight traits, namely, PH, GN, SS, YPP, HD, EPN, TGW, and PL, five QTLs were identified. GBS, a economical alternative to other whole-genome genotyping platforms, allowed us to calculate the substituted fragment of parental inbred lines accurately. We directly evaluated the agronomic traits of the F_1_ population derived from 156 CSSLs in the field. The *qHD8*, with a 214.46 kb region, contains the previously reported gene *RH8*/*DTH8* (Gao et al. 2013; Li et al. 2016).

The China Rice Data Center database (http://www.ricedata.cn/) placed *pms1* at approximately 6.69 Mb on chromosome 7, ~ 2.19 Mb from *qSS7* (4.27–4.50 Mb). The QTL *qSS7* could be a unique gene rather than *pms1*. If this is true, its isolation could clarify regulation of the rate of seed setting, a key yield component in hybrid rice.

Male sterility of the parent PA64s is conferred by photoperiod and thermo-sensitive male sterile genes. LOC_Os07g12130 is the thermo-sensitive candidate gene for *pms1* (Zhou et al. 2011a, b). The quantitative traits are influenced by multiple genes or QTLs (Li et al. 2003). Gene expression could be greatly affected by environmental factors (Cao et al. 2001). In this study, the distance between *qSS7* and *pms1* could be due to environmental effects on the mapping of *qSS7*. Although the *pms1* gene was previously reported to control pollen male sterility, we found that *qSS7* increased the rate of seed setting and reduced plant height.

The *pms1* gene encodes a long-non-coding RNA *PMS1T* that was reported to be preferentially transcribed in young panicles. *PMS1T* was shown to be a long non-coding RNA (lncRNA), targeted by miR2118 to produce 21-nt phasiRNAs. Under long-day conditions, these phasiRNAs preferentially accumulate in photoperiod-sensitive male sterile rice genotypes, and the higher accumulation of phasiRNAs is the cause of male sterility (Fan et al. 2016). Non-coding RNAs have been found to function in a wide range of plant physiological mechanisms. For example, phasiRNAs preferentially accumulate in maize reproductive tissues (Zhai et al. 2015). It will be intriguing to determine whether *pms1* underlying male sterility is the candidate gene in *qSS7* that controls the rate of seed setting and plant height. As the heterozygous segments harboring the heterosis genes affect yield in hybrid rice, the CSSLs harboring these genes can be used as donor parental lines for heterosis utilization.

## Electronic supplementary material

Below is the link to the electronic supplementary material.

Supplementary material 1 (PDF 1728 kb)
