# Supplementary material for: Heterosis-associated genes confer high yield in super hybrid rice
Source: Theor Appl Genet. 2020 Aug 27;133(12):3287–97. doi: 10.1007/s00122-020-03669-y (PMC7567734; doi:10.1007/s00122-020-03669-y)
Supplement: Supplementary file 1 — Supplementary material 1 (PDF 1728 kb) [file 122_2020_3669_MOESM1_ESM.pdf]

Supplementary materials

A

CSSLs×9311 F_1 vs CSSLs

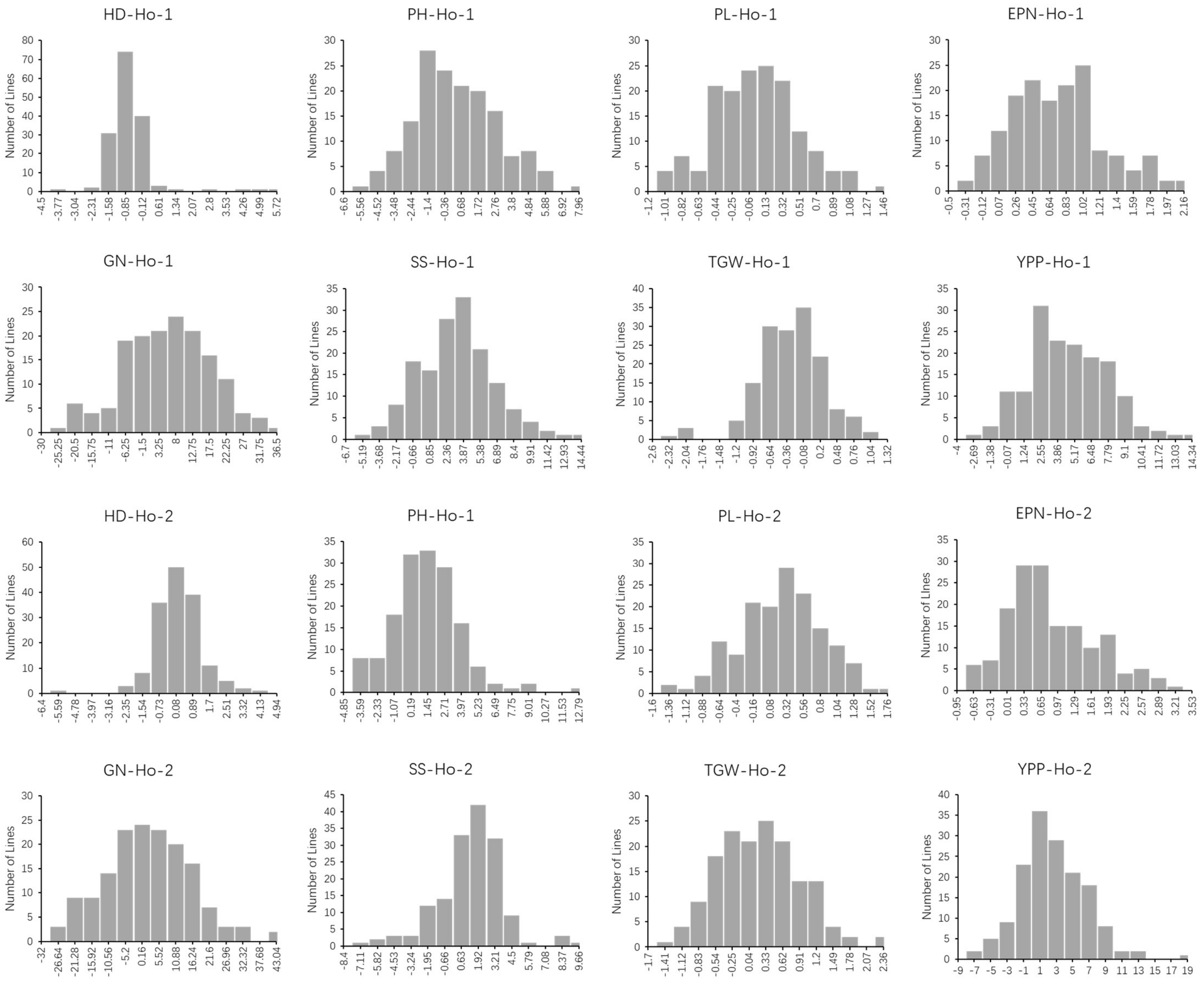

B

PA64s×CSSLs vs PA64s ×9311

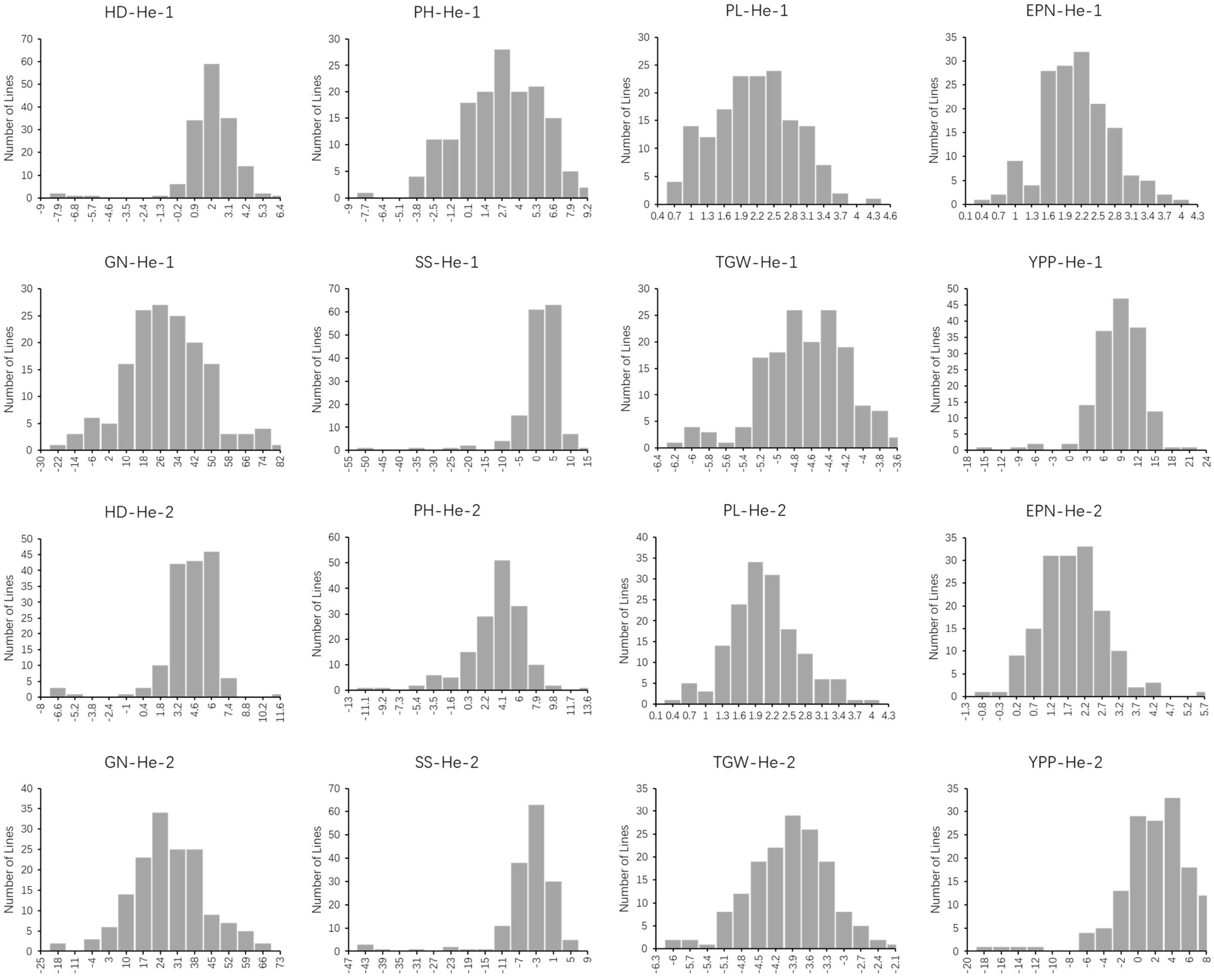

Fig. S1 Histograms of frequency distributions.

(A) Values of mid-parent heterosis in a homozygous background, (B) values of over-standard heterosis in a heterozygous background.

Mid-parent heterosis was calculated as CSSLs / 9311 – CSSLs; Over-standard heterosis was calculated as PA64s / CSSLs – PA64s / 9311.

HD, heading date; PH, plant height; PL, panicle length; EPN, effective panicle length; GN, grain number per panicle; SS, rate of seed setting; TGW, 1,000-grain weight; YPP, yield per plant.

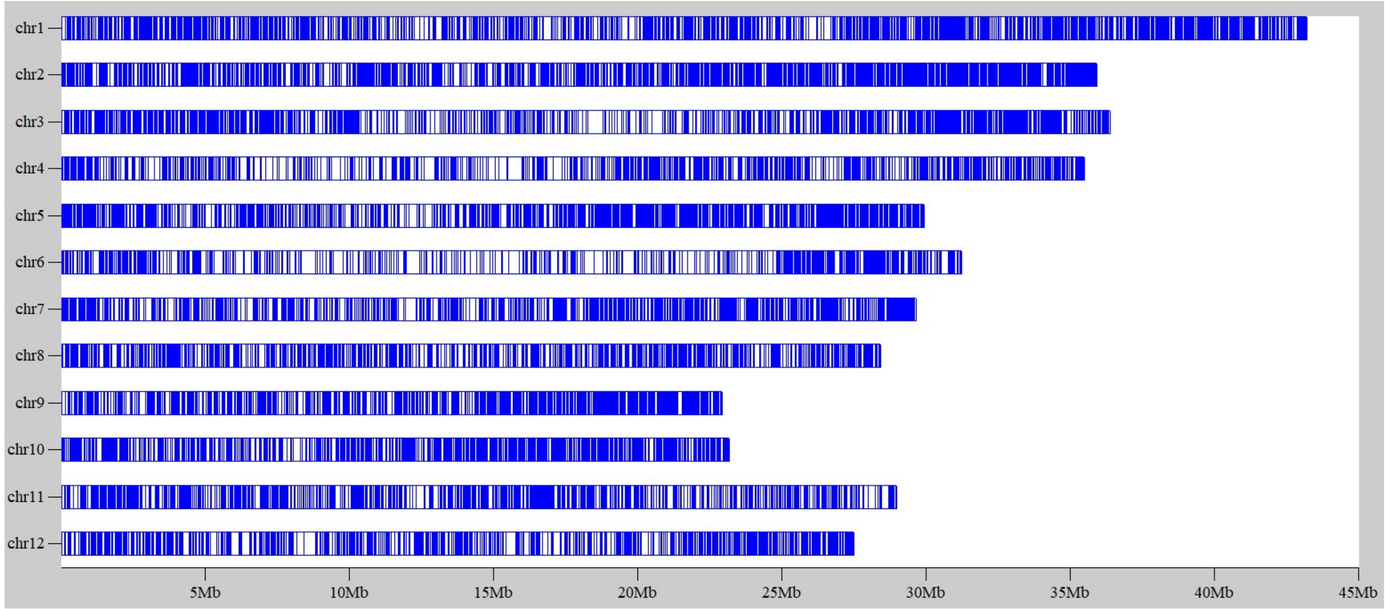

Fig. S2 Genome-wide distribution of the 10,768 SNPs identified by genotyping–by–sequencing (GBS).

SNPs were detected and labeled in blue regions, while no SNP were detected and labeled in white regions.

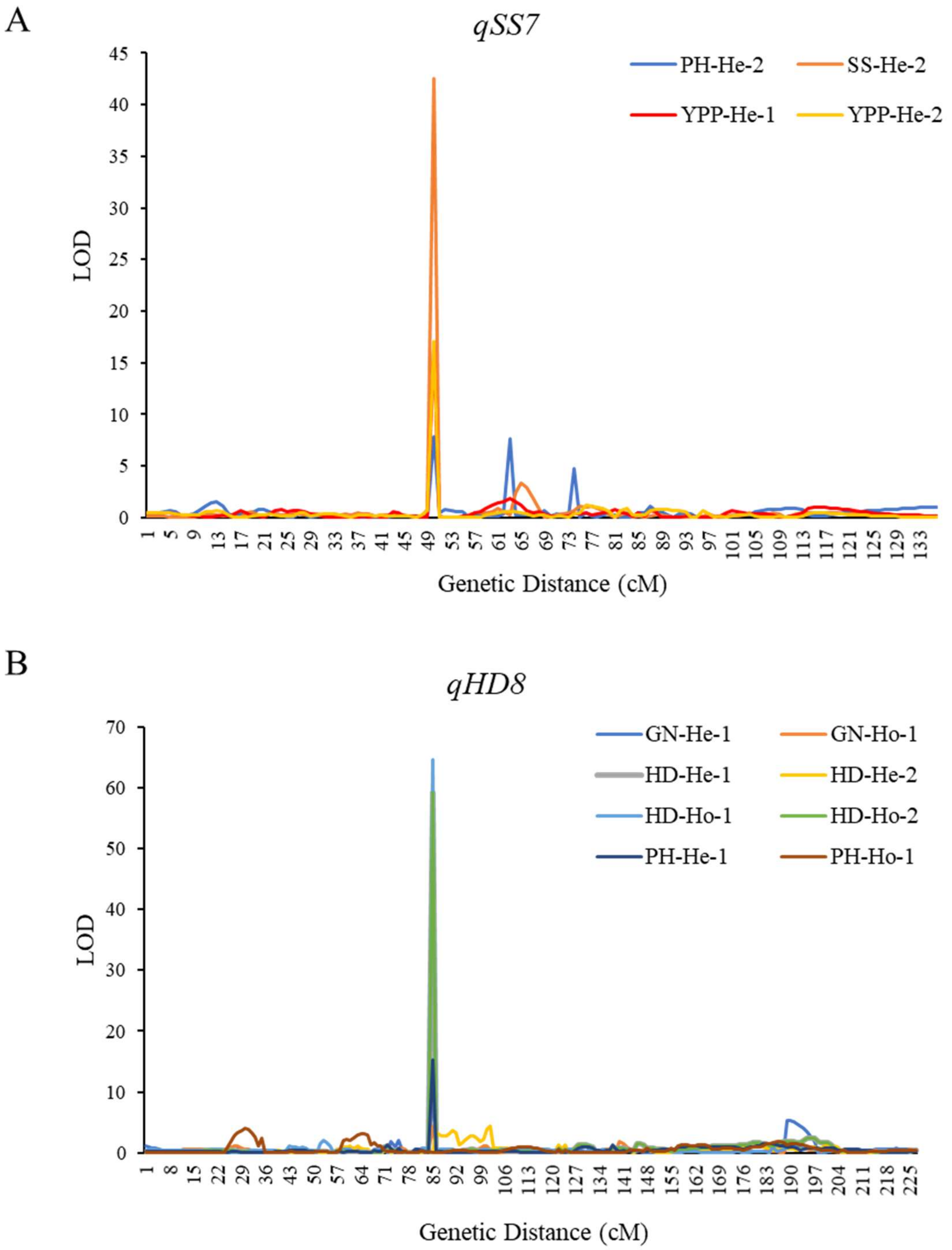

Fig. S3 The two QTLs *qSS7* and *qHD8* were repeatedly detected in the two environments. The X-axis corresponds to the genetic map in centimorgans, and the Y-axis corresponds to the LOD value as calculated by ICIM. Two QTLs were detected on the two chromosomes 7 and 8, respectively. The QTL *qSS7* was located between the left marker 4,272,700 and the right marker 4,499,836 (LOD = 42.51) on Chr 7. The other QTL *qHD8* was located at the left marker 4,094,492 and the right marker 4,308,948 (LOD = 64.59) on Chr 8.

A. The *qSS7* was detected to underlie the traits of SS-He-2, PH-He-2, YPP-He-1 and YPP-He-2.

B. The *qHD8* was detected to underlie the traits of GN-He-1, GN-Ho-1, HD-He-1, HD-He-2, HD-

Ho-1, HD-Ho-2, PH-He-1 and PH-Ho-2.

Table S1 Phenotypic variation of the eight yield-related traits in the CSSLs and the two F_1 hybrids in two environments.

Trait	Environ-ment	9311	CSSLs				B-homo F_1 hybrids					LYP9	B-heter F_1 hybrids				
			Mean	SD	Min	Max	Mean	SD	Min	Max	MPH		Mean	SD	Min	Max	ES
HD	E1	101.86	102.86	2.76	116.50	88.50	101.60	1.08	109.50	98.50	0.58	106.13	104.03	2.94	109.50	87.00	-1.98
	E2	100.75	100.42	2.84	115.00	89.00	100.28	1.54	109.00	93.00	-0.31	105.50	104.96	3.23	110.00	88.00	-0.52
PH	E1	130.12	129.41	5.39	142.19	107.39	128.87	3.44	137.13	121.38	1.52	132.34	130.84	3.87	137.35	116.90	-1.13
	E2	132.85	132.72	5.15	143.68	112.14	133.51	2.75	140.57	125.58	0.73	129.61	135.21	3.93	142.91	119.03	1.00
PL	E1	23.12	23.10	0.82	26.12	20.81	22.94	0.60	25.04	21.77	0.19	25.51	25.03	0.78	27.11	23.35	-1.87
	E2	22.65	23.18	0.91	26.60	21.12	23.03	0.67	24.79	21.32	0.12	25.13	24.92	0.57	26.37	23.34	-0.86
GN	E1	209.43	205.94	17.32	243.78	153.09	211.55	11.84	239.84	182.48	5.06	224.01	232.92	19.92	284.17	165.99	4.03
	E2	191.98	190.71	16.01	234.86	149.93	191.00	13.52	238.26	160.49	-0.35	216.51	215.54	16.02	269.73	161.99	-0.45
EPN	E1	5.00	5.00	0.41	6.00	3.92	5.59	0.54	7.08	4.50	0.50	6.90	6.92	0.61	8.83	5.06	0.45
	E2	6.75	6.99	0.73	8.81	5.20	7.55	0.83	9.70	5.89	0.68	8.12	8.42	0.95	12.50	5.50	3.71
SS	E1	75.50	79.86	5.83	89.66	60.03	80.95	3.73	91.85	70.42	3.61	78.12	76.84	8.15	88.79	22.06	-1.57
	E2	91.43	90.56	3.07	95.34	79.44	91.68	2.22	95.27	80.03	0.68	84.18	83.91	7.89	93.37	45.11	-0.32
TGW	E1	30.33	29.88	1.23	32.79	25.28	29.56	0.83	31.98	27.20	0.59	26.43	25.23	0.68	27.50	23.08	-4.56
	E2	29.54	29.23	1.26	32.90	24.97	29.46	0.80	31.77	27.25	0.07	25.22	25.26	0.80	27.11	22.63	0.18
YPP	E1	23.81	24.56	2.89	31.85	16.60	28.15	2.91	36.01	18.73	3.48	32.59	31.14	4.68	40.03	7.18	-4.42
	E2	37.80	35.90	4.21	45.05	16.46	38.48	3.90	54.13	27.89	1.63	40.31	38.18	4.65	48.81	17.50	-5.30

YPP, yield per plant; GN, grains per panicle; TGW 1000-grain weight; EPN, effective panicle number; PH, plant height; HD, heading date; PL, panicle length; SS, rate of seed setting; CV, Coefficient of variation, given by SD/mean×100%; SD, standard deviation of mean value; MPH mid-parent heterosis, given by F_1-(CSSLs + 9311)/2 in the B-homo F_1 set; ES, effects by substituted segment, by F_1/ LYP9 ×100%-100 in the B-heter F_1 set.

Table S2 Phenotypic correlations of the eight yield-related traits between CSSLs and the B-homo F_1 set (left) and between CSSLs and the B-heter F_1 set (right).

Set	Trait	B-homo F_1								B-heter F_1							
		HD	PH	PL	GN	EPN	SS	TGW	YPP	HD	PH	PL	GN	EPN	SS	TGW	YPP
**CSSLs**	HD	0.74**	0.17*	0.16*	0.09	-0.08	-0.23**	0.14	0.04	0.85**	0.20*	-0.06	0.26**	-0.19*	0.03	0.28**	0.16*
	PH	0.16*	0.65**	0.12	-0.09	-0.01	-0.21**	0.27**	0.05	0.54**	0.67**	0.1	0.33**	-0.11	0.18*	0.50**	0.37**
	PL	0.15	0.31**	0.67**	0.04	-0.01	0.05	0.24**	0.07	-0.07	0.01	0.38**	0.09	-0.17*	0.17*	-0.01	0.11
	GN	0.03	0.07	0.00	0.32**	-0.12	-0.11	-0.24**	0.04	0.23**	0.09	-0.09	0.37**	-0.15	-0.10	-0.03	0.01
	EPN	0.00	0.02	-0.09	-0.13	0.21**	-0.10	-0.17*	-0.01	-0.13	-0.01	-0.02	-0.16*	0.09	-0.02	-0.20*	-0.05
	SS	0.1	0.03	0.04	-0.03	-0.01	0.32**	0.03	0.02	0.08	0.21**	0.08	0.00	-0.02	0.45**	0.03	0.37**
	TGW	0.20*	0.40**	0.26**	-0.12	-0.1	-0.01	0.71**	0.14	0.32**	0.35**	0.19*	-0.01	-0.07	0.19*	0.70**	0.26**
	YPP	0.14	0.32**	0.14	0.12	-0.02	-0.02	0.05	0.11	0.38**	0.35**	-0.08	0.22**	-0.12	0.11	0.22**	0.20*

The eight yield-related traits are as follows. YPP, grain weight per plant; GN, grain number per panicle; TGW, 1000-grain weight; EPN, effective panicle number; PH, plant height; HD, heading date; PL, panicle length; SS, rate of seed setting;

*, **, significant at $P < 0.05$ and 0.01, respectively.

Table S3 Analysis of variance on the yield-related traits in the CSSLs and the two F_1 hybrids sets.

Trait[a]	CSSLs population			B-homo F_1 set			B-heter F_1 set		
	Lines	E[C]	Lines×E	Lines	E	Lines×E	Lines	E	Lines×E
DF[b]	156	1	156	156	1	156	156	1	156
HD	1648.14**	51.04**	4.82**	1056.7**	22.37**	4.84**	247.77**	65.24**	4.91**
PH	492.08**	27.2**	4.75**	1022.72**	6.67**	5.18**	508.25**	8.55**	1.81**
PL	4.88*	11.31**	2.73**	7.46**	6.74**	3.16**	6.90**	3.74**	3.07**
GN	269.75**	5.84**	2.45**	657.46**	3.53**	2.91**	243.06**	4.37**	2.37**
EPN	2944.18**	3.85**	2.90**	1967.4**	2.83**	3.59**	852.47**	3.33**	2.91**
SS	2744.61**	6.11**	5.33**	4752.53**	4.26**	4.72**	1105.92**	24.38**	4.05**
TGW	288.49**	24.72**	3.00**	8.82**	10.77**	3.11**	1.61	15.65**	4.56**
YPP	3833.88**	6.07**	3.89**	3293.36**	4.66**	4.70**	1070.07**	8.77**	3.29**

YPP, yield per plant; GN grain number per panicle; TGW 1000-grain weight; EPN, effective panicle number; PH, plant height; HD heading date; PL, panicle length; SS, rate of seed setting; [b]DF, degrees of freedom; [c] E: Environments 2. *, ** Significant at $P < 0.05$ and 0.01, respectively.

Table S4 QTLs identified in the homozygous background (Ho).

QTL	Trait name	Chr	Position	Left marker	Right marker	LOD	PVE (%)	Add	Dom	$\left\|\frac{d}{a}\right\|$	Dominance degree
qHD1	HD-Ho-1	1	122	217,210	334,059	19.12	6.757	-2.527	1.223	0.484	PD
qHD7.1	HD-Ho-1	7	44	15,242,593	15,359,676	42.30	22.246	2.876	-2.110	0.734	PD
qHD7.2	HD-Ho-2	7	46	7,996,783	8,915,889	5.22	2.728	-1.185	-0.794	0.670	PD
	HD-Ho-1	8	85	4,094,492	4,308,948	64.60	51.300	6.096	-5.893	0.967	CD
qHD8	HD-Ho-2	8	85	4,094,492	4,308,948	59.16	76.477	-8.075	7.525	0.932	CD
	PH-Ho-1	8	85	4,094,492	4,308,948	14.66	17.083	7.215	-10.430	1.446	OD
qPH1	PH-Ho-2	1	175	32,814,522	33,788,488	7.52	7.221	-2.915	3.757	1.289	OD
qPH3	PH-Ho-2	3	25	21,702,272	26,785,589	13.09	14.938	7.838	-7.028	0.897	CD
qPH8	PH-Ho-2	8	85	4,094,492	4,308,948	23.21	28.625	7.724	-9.389	1.216	OD
qPH11	PH-Ho-2	11	65	14,649,799	20,624,633	5.25	5.459	1.801	-5.835	3.240	OD
qEPN9	EPN-Ho-1	9	103	3,806,405	18,923,154	5.48	9.245	0.115	1.132	9.821	OD
qSS8.1	SS-Ho-1	8	186	5,978,133	9,092,592	7.23	16.972	2.846	3.230	1.135	CD
qSS8.2	SS-Ho-2	8	71	5,383,861	20,995,010	6.78	3.542	6.172	-5.396	0.874	CD
qTGW1	TGW-Ho-1	1	12	3,430,885	14,714,228	6.20	8.507	1.939	-2.101	1.083	CD

The QTL *qHD8* was repeatedly identified to underlie the traits of HD-Ho-1, HD-Ho-2 and PH-Ho-1.

HD, heading date; PH, plant height; EPN, effective panicle number; SS, rate of seed setting; TGW, 1,000-grain weight; LOD, log of odds; PVE, phenotypic variation explained; Add, additive effect; Dom, dominance effect; |d/a|, dominance ratio, the absolute value of dominance effect to additive effect; PD, partial dominance; CD, complete dominance; OD over-dominance

Table S5 QTLs identified in the heterozygous background (He).

QTL	Trait name	Chr	Position	Left marker	Right marker	LOD	PVE (%)	Add	Dom
qHD7	HD-He-1	7	46	7,996,783	8,915,889	5.22	2.728	-1.185	-0.794
qHD8	HD-He-1	8	85	4,094,492	4,308,948	59.16	76.477	-8.075	7.525
	HD-He-2	8	85	4,094,492	4,308,948	43.75	43.303	-8.367	8.258
qPH5	PH-He-1	5	89	21,474,218	25,325,589	5.68	8.043	-4.291	4.041
qPH6	PH-He-1	6	5	26,412,872	26,593,549	10.2	14.098	-4.424	5.149
	PH-He-2	6	5	26,412,872	26,593,549	16.46	19	-5.509	7.79
qSS7.1	PH-He-2	7	50	4,272,700	4,499,836	7.83	7.9	-4.544	-5.078
qPH7	PH-He-2	7	63	7,182,159	7,182,195	7.59	7.673	-3.381	-5.315
qHD8	PH-He-1	8	85	4,094,492	4,308,948	15.25	22.828	-6.261	1.453
qPH9	PH-He-2	9	62	8,389,412	18,893,636	9.16	9.6	1.519	-18.274
qEPN4	EPN-He-2	4	76	12,321,968	21,535,281	5.14	2.91	1.46	-2.08
qSS3.1	SS-He-2	3	81	4,583,739	22,413,887	7.23	5.101	-10.749	6.229
qSS3.2	SS-He-1	3	67	10,291,020	10,618,830	5.27	2.774	-7.582	0.628
qSS4	SS-He-1	4	65	17,868,628	18,154,552	11.38	5.977	-8.551	7.678
	SS-He-2	4	65	17,868,628	18,154,552	9.62	6.546	-8.795	7.43
qSS6	SS-He-2	6	32	4,367,595	4,813,022	6.88	4.435	-6.011	-3.728
qSS7.1	SS-He-2	7	50	4,272,700	4,499,836	42.51	49.647	-19.877	19.032

qSS7.2	SS-He-1	7	49	5,263,738	8,524,035	51.45	58.961	-20.626	1.01
qSS9	SS-He-1	9	65	21,383,583	21,652,040	15.25	8.731	-12.741	-1.542
	SS-He-2	9	65	21,383,583	21,652,040	10.4	7.417	-9.078	1.352
qSS10	SS-He-1	10	191	12,060,160	12,301,639	5.84	2.819	3.541	1.378
qHD8	TGW-He-2	8	85	4,094,492	4,308,948	7.1	13.084	-1.049	2.203
	GN-He-1	8	85	4,094,492	4,308,948	9.91	16.63	-27.897	34.233
qGN8	GN-He-1	8	190	9,082,002	9,092,592	5.29	8.184	-10.642	-2.734
qYPP3	YPP-He-2	3	3	877,165	4,583,739	9.03	12.704	-10.618	14.857
qSS7.1	YPP-He-1	7	50	4,272,700	4,499,836	16.71	33.341	-9.788	9.402
	YPP-He-2	7	50	4,272,700	4,499,836	17.02	26.124	-8.987	8.635
qYPP9	YPP-He-1	9	18	17,400,253	22,881,257	5.3	8.772	-3.331	1.739

The QTL *qSS7.1* was repeatedly identified to underlie the traits of PH-He-2, SS-He-2, YPP-He-1 and YPP-He-2, while *qHD8* was repeatedly identified to underlie the traits of HD-He-1, HD-He-2, PH-He-1, TGW-He-2 and GN-He-1.

YPP, yield per plant; EPN, effective panicle number; SS, rate of seed setting; PH, plant height; HD, heading date; GN, grain number per plant; TGW, 1,000-grain weight; LOD, log of odds; PVE, phenotypic variation explained; Add, additive effect; Dom, dominance effect.

Table S6 F tests for the two QTLs *qSS7* and *qHD8* underlying YPP and YPP-related traits in the B-heter F_1 set.

Trait	SNP difference		Error		*F* value	*P* value
	DF	MS	DF.	MS		
HD-1	2	384.87	152	1.81	212.42	<0.0001
HD-2	2	427.91	152	3.25	131.73	<0.0001
PH-1	2	215.97	152	11.47	18.82	<0.0001
PH-2	2	152.22	152	13.60	11.19	<0.0001
PL-1	2	3.00	152	0.57	5.26	0.0062
GN-1	2	4123.90	152	320.76	12.86	<0.0001
SS-1	2	1432.61	152	48.35	29.63	<0.0001
SS-2	2	1485.52	152	43.76	33.95	<0.0001
TGW-2	2	8.05	152	0.52	15.39	<0.0001
YPP-1	2	329.83	152	17.72	18.61	<0.0001
YPP-2	2	340.60	152	17.36	19.62	<0.0001

DF, degrees of freedom; MS, mean square; SNP difference, *qSS7*$^{\mathrm{PA64s/9311}}$/*qHD8*$^{\mathrm{PA64s/9311}}$, *qSS7*$^{\mathrm{PA64s/9311}}$/*qHD8*$^{\mathrm{PA64s/PA64s}}$, *qSS7*$^{\mathrm{PA64s/PA64s}}$/*qHD8*$^{\mathrm{PA64s/9311}}$; HD, heading date; PH, plant height; PL, panicle length; GN, grain number per plant; SS, rate of seed setting; TGW, 1,000-grain weight; YPP, yield per plant.
